# Effect and clinical mechanism exploration of acupuncture intervention for chemotherapy-related cognitive impairment (CRCI) in triple-negative breast cancer: study protocol for a randomized controlled trial

**DOI:** 10.3389/fneur.2025.1565040

**Published:** 2025-07-23

**Authors:** Wenqi Yang, Qing Zhang, Chong Gao, Jingzhi Zhang, Xingwei Guo, Xin Liu

**Affiliations:** ^1^Graduate School, Beijing University of Chinese Medicine, Beijing, China; ^2^Department of Oncology, Beijing Hospital of Traditional Chinese Medicine, Capital Medical University, Beijing, China; ^3^Graduate School, Capital Medical University, Beijing, China

**Keywords:** chemotherapy-related cognitive impairment, triple-negative breast cancer, cytokine, acupuncture, sham acupuncture, clinical research trial

## Abstract

**Background:**

Chemotherapy-Related Cognitive Impairment (CRCI) significantly impacts the quality of life of breast cancer patients. Triple-negative breast cancer (TNBC) is associated with a higher risk of cognitive decline. The occurrence of CRCI is linked to the expression of inflammatory cytokines. Currently, limited research has examined the efficacy of acupuncture for treating CRCI in TNBC patients. This randomized controlled trial aims to evaluate the effectiveness of acupuncture in managing CRCI among TNBC patients and explore the mechanism by which acupuncture treatment affects CRCI through the inflammatory signaling pathway.

**Methods:**

This study is designed as a prospective, parallel, randomized, sham-controlled, assessor-blinded clinical trial. It will involve 50 patients diagnosed with TNBC who also experience CRCI. Participants will be randomly assigned to two groups, with an equal 1:1 allocation ratio into either the intervention group or the control group. Both groups will receive acupuncture sessions twice weekly for 8 weeks, with each session lasting approximately 20 min. The primary outcome of this study will be the percentage of subjects showing improvement in the Montreal Cognitive Assessment (MoCA) score at the end of treatment. Secondary outcome measures will include the Mini-Mental State Examination (MMSE) score, EORTC QLQ-C30 score, and the expression of inflammatory cytokines.

**Discussion:**

The findings of this study are expected to provide additional evidence supporting the efficacy of acupuncture and contribute clinical data that may elucidate the potential therapeutic mechanisms by which acupuncture ameliorates CRCI.

**Trial registration:**

https://www.chictr.org.cn/showproj.html?proj=218356, identifier: ChiCTR2400080147.

## 1 Introduction

Breast cancer remains the primary cause of elevated cancer morbidity and disability among women (1). More than 50% of breast cancer patients undergo chemotherapy treatment (2), with up to 81.4% of patients in China requiring adjuvant chemotherapy (3). Chemotherapy-Related Cognitive Impairment (CRCI) is a common clinical complication in breast cancer survivors. The clinical manifestations encompass a spectrum of neurocognitive symptoms characterized by diminished cognitive function, evidenced by subtle declines in memory, mental clarity, and the capacity to maintain focus or organization during or after chemotherapy (4). Estimates suggest that approximately 75% of patients experience cognitive impairment during chemotherapy, while 35% continue to experience cognitive impairment and a reduced quality of life for several years post-chemotherapy (5). The 5-year relative survival rate for breast cancer patients has improved from 75 to 90% (2). However, CRCI could impede these patients’ reintegration into the workforce. This condition may interfere with adherence to treatment regimens and impact patients’ ability to enjoy an enhanced quality of life and social functioning, potentially leading to further economic consequences (6–8).

Triple-negative breast cancer (TNBC) is a significant risk factor for CRCI. Patients with TNBC exhibit more severe overall cognitive dysfunction and memory impairment compared to non-TNBC patients. Liu Z et al. (9) demonstrated a correlation between hormone receptor and HER2 status and CRCI in breast cancer patients, potentially due to the protective effects of these receptors on cognitive function. Additional research (10) indicates that patients with estrogen receptor (ER) and progesterone receptor (PR) double-negative status are more susceptible to cognitive decline and memory impairment following chemotherapy, compared to those with ER and PR positive status. These observations underscore the strong association between cognitive impairment and hormone receptor status.

Despite substantial evidence indicating the high prevalence of CRCI, there is a notable scarcity of therapies specifically developed to address this condition. Pharmacological interventions have shown no efficacy in preventing or treating CRCI (11). Furthermore, the cognitive benefits of cognitive training and physical activity interventions for individuals experiencing cognitive problems remain ambiguous, and the effectiveness of these interventions requires further demonstration (11, 12).

Acupuncture, a fundamental component of traditional Chinese medicine (TCM), has been utilized for millennia in treating various neurological disorders. Multiple systematic reviews and meta-analyses have demonstrated the efficacy and safety of acupuncture in treating mild cognitive impairment when compared to medication, usual care, and sham acupuncture (13–19). Taishan Tong et al. (19) reported the application of acupuncture therapy for CRCI in breast cancer patients, albeit without follow-up observations. Our previous research (17, 18) also indicated that acupuncture influences overall cognitive function in patients with CRCI, potentially offering complementary therapeutic options. However, current studies lack robust methodological evidence, and the mechanisms underlying acupuncture’s effects remain subject to debate.

Prior research has demonstrated a correlation between the occurrence of CRCI and inflammatory processes, including the activity of cytokines and lymphocytes (20–24). Among the factors contributing to cognitive deficits, oxidative stress and inflammation are considered to be potentially the most significant (25–27). Cytokines are believed to influence cognitive functions through various mechanisms, including the modulation of immune responses, synaptic plasticity, neurotrophic support, neuroinflammatory processes, the maintenance of blood–brain barrier (BBB) integrity, and the regulation of gene expression related to cognition (28).

To the best of our knowledge, cytokines may play a crucial role in the pathogenesis of CRCI. Previous studies have shown that cytokines show different temporal trends during and after chemotherapy, and are associated with specific areas of cognitive impairment (29, 30). IL-6 levels show a biphasic pattern, with consistently increasing concentrations from the start of chemotherapy to 6 months post-chemotherapy, followed by a gradual decline over time after 6 months (29). In contrast, IL-1β and IL-4 levels remain significantly elevated after chemotherapy and are associated with long-term deficits in verbal memory and executive function (30, 31). IL-7 and IL-10 show transient suppression early in the course of chemotherapy, with recovery trajectories associated with improved verbal fluency (30). Several scholars have proposed that a quantitative cytokine level balance or ratio is also associated with the development of CRCI, that cytokines present at different concentration levels in different regions of the brain, and that memory function correlates with optimal concentration levels of specific cytokines (30). TNF-*α* is associated with acute cognitive trajectories (32), and IL-6 and IL-8 are associated with persistent cognitive trajectories (33). Elevated concentrations of IL-6 and IL-1β may result in a decrease in reaction speed and perceptual functioning (33). IL-4 has a protective effect on reaction speed and the higher the concentration, the better the learning ability (32).

However, no study has yet been conducted to elucidate the underlying mechanisms of acupuncture for CRCI based on cytokine activity. Therefore, we will conduct a randomized controlled clinical trial to evaluate the efficacy and safety of acupuncture for the treatment of CRCI in patients with TNBC. This study aims to provide high-level evidence supporting the potential therapeutic mechanisms of acupuncture. Specifically, we seek to elucidate the therapeutic mechanisms of acupuncture for CRCI by examining the expression levels of inflammatory cytokines.

## 2 Methods and analysis

### 2.1 Design and setting

**Figure 1:**
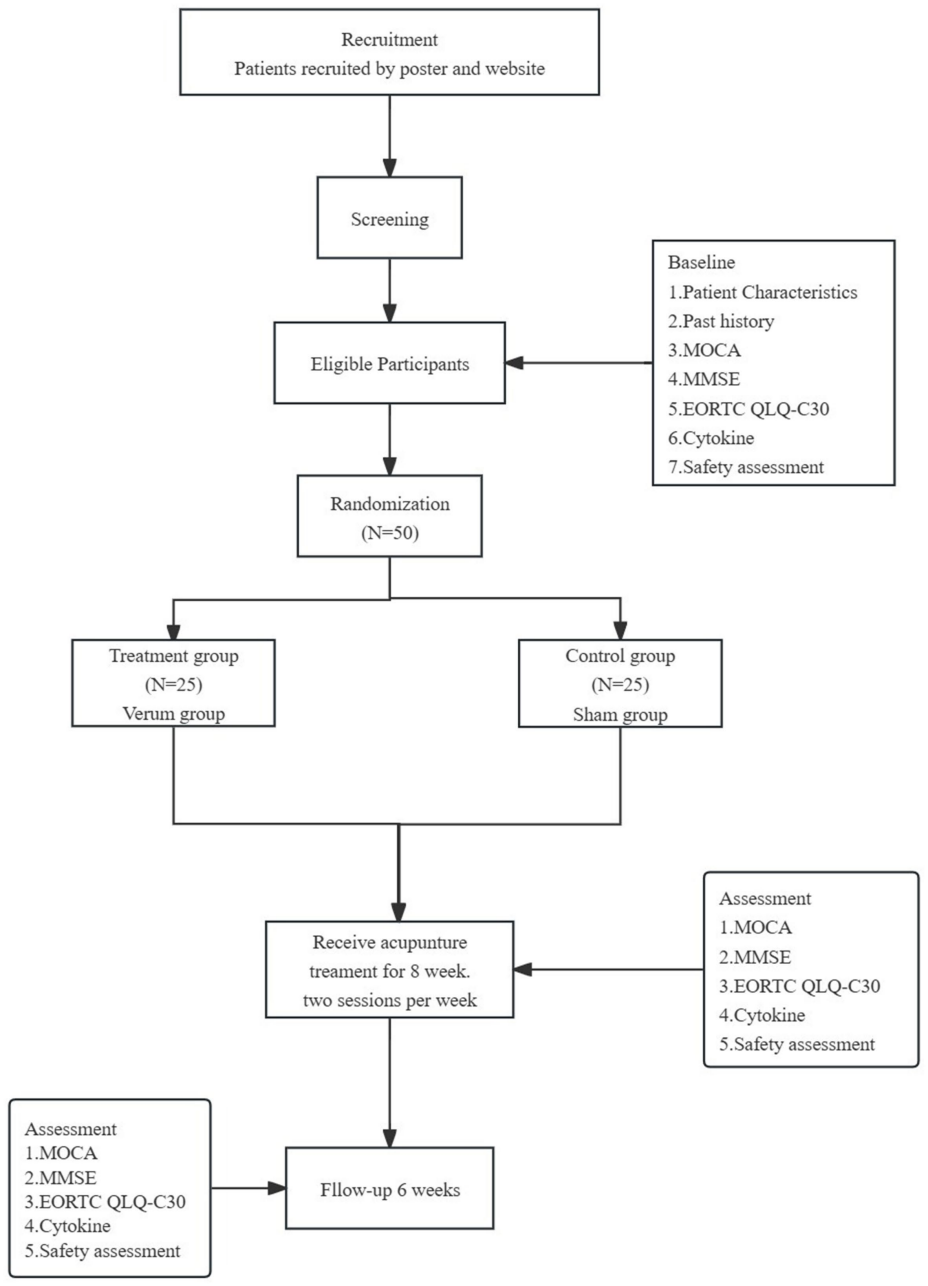
Project overview. MOCA, Montreal Cognitive Assessment; MMSE, Mini-Mental State Examination; EORTC QLQ-C30, The European Organization for Research and Treatment of Cancer QLQ-C30; Expression of inflammatory cytokines (TNF-*α*, IL-1β, IL-2, IL-4, IL-5, IL-6, IL-8, IL-10, IL-12p70, IL-17, IFN-*γ*, IFN-α).

A parallel, randomized, sham-controlled prospective trial will be conducted in Beijing Hospital of Traditional Chinese Medicine, Capital Medical University. This study is designed as a parallel, single-center, randomized, sham-controlled prospective experiment to evaluate the efficacy of acupuncture treatment for triple-negative breast cancer patients experiencing chemotherapy-induced cognitive impairment. The trial will be conducted from January 22, 2024 to May 1, 2026 at Beijing Hospital of Traditional Chinese Medicine, Capital Medical University. Eligible patients with CRCI will be randomly allocated to either the verum acupuncture group or the sham acupuncture group in a 1:1 ratio. The observation period spans 14 weeks, comprising an 8-week treatment phase followed by a 6-week follow-up phase. Figure 1 illustrates the flowchart of the study procedure, while Table 1 outlines the assessment time points.

**Table 1:** Schedule of enrollment, interventions, and assessments.

Time point	Baseline	Treatment phase			Follow-up phase
	Week 0	Week 1	→	Week 8	Week 14
Enrollment					
Eligibility screen	×				
Informed consent	×				
Randomization	×				
Interventions					
Verum acupuncture		16 sessions treatment			
Sham acupuncture		16 sessions treatment			
Laboratory test					
Cytokine	×			×	×
Assessments					
MOCA	×			×	×
MMSE	×			×	×
EORTC QLQ-C30	×			×	×
Adverse events		×	→	×	

### 2.2 Patient

#### 2.2.1 Recruitment strategies

Recruitment of participants will occur through outpatient clinics and wards at Beijing Hospital of Traditional Chinese Medicine, Capital Medical University, utilizing advertisements on posters and via WeChat to ensure an adequate sample size. Research assistants will be deployed to the hospitals to conduct participant screening.

Prior to commencing treatment, participants will undergo a comprehensive baseline assessment, and their cancer history will be meticulously documented. This documentation encompasses details of previous surgeries, pathology reports, and information regarding prior treatments. Additionally, a thorough safety evaluation will be conducted.

#### 2.2.2 Inclusion criteria

1. Patients with histologically, cytologically, or radiographically confirmed triple-negative breast cancer.
2. Age 18–65 years.
3. Patients exhibiting cognitive impairment within 3 years post-chemotherapy and those diagnosed with mild cognitive impairment.
4. The syndrome differentiation according to TCM is of the Qi-Xue-Shi-Tiao type.
5. Patients are expected to have a life expectancy exceeding 1 year.
6. Participants voluntarily enrolled in the study and provided written informed consent.

#### 2.2.3 Exclusion criteria

1. Cognitive dysfunction secondary to other conditions, such as central nervous system infections, head trauma, stroke, etc.
2. Patients who have been taking medications that affect cognition within the past 3 months; or patients with a history of alcohol dependence or abuse of other psychoactive substances.
3. Patients with severe visual acuity deficits, hearing impairment, severe aphasia, or other significant diagnoses.
4. Severe internal diseases or central nervous system disorders, including but not limited to severe anemia.
5. Patients with brain metastases, cerebral hemorrhage, cerebral thrombosis, traumatic brain injury, or severe cardiovascular disease.
6. Patients who have undergone cognitive rehabilitation within the previous 3 months.
7. Patients who are unable to independently complete the relevant scale due to other factors.

### 2.3 Randomization and blinding

A computer-generated block randomization with 1:1 allocation will be used to assign 50 eligible participants. An independent statistician at the Beijing Hospital of Traditional Chinese Medicine, Capital Medical University will implement the randomization procedure using SPSS 25.0 statistical software (IBM Corporation, Armonk, NY, United States), employing randomly permuted blocks to ensure allocation concealment. The allocation information will become available only after the randomization procedure. Subsequently, a second individual will employ an opaque envelope containing a randomly sorted distribution card to assign patients to either the verum acupuncture group or the sham acupuncture group, based on their hospital visits. The research assistant responsible for randomization and acupuncturists are not blinded to the participant allocation. The data analysts and outcome assessors will not be aware of the allocated group.

To mitigate potential expectations regarding acupuncture treatment, patients will be informed that they will randomly receive one of two different acupuncture regimens. It is not possible to blind participants, owing to the different acupuncture points of the two groups. To minimize potential communication and interaction among participants, each patient will receive individualized acupuncture treatment and follow-up at separate times in distinct rooms. Due to the inherent nature of acupuncture intervention, blinding the acupuncturist is not feasible. Blinding will be applied to the outcome assessment. Data analysts and outcome evaluators will be blinded to the treatment allocation. Furthermore, to maintain the integrity of the study, communication between acupuncturists, participants, and outcome evaluators will be carefully structured to prevent any exchange of information. The assessor will be arranged to conduct assessment on different days from acupuncture treatment to avoid cross communication among the assessor, acupuncturists, and participants. The assessor will also be instructed not to acquire participants’ treatment information and their needling feelings. Prior to analysis, all data will be anonymized to ensure objectivity.

### 2.4 Written informed consent

For any TNBC participants who meet the inclusion and exclusion criteria, they will be approached to participate in the study by one of the investigators from the study team. All participants will be provided with a written copy of the consent form detailing their random assignment to either verum acupuncture or sham acupuncture, as well as the potential benefits and risks. The investigator will be responsible for ensuring that the patient completely understands the potential risks and benefits of participating in the study and for answering any questions the patient may have. Responses will be in a language that the subject understands, with translation and visual aids as necessary. Participants can contact the investigator at any time to inform them of their decision and to ask any questions about the study through outpatient or inpatient visits, email contact, phone contact, WeChat contact or other web-based contacts. Prior to participating in the research, all participants will voluntarily sign the informed consent form. Withdrawal conditions: (1) Participants may choose to withdraw at any stage of the trial if they prefer not to receive the assigned treatment. (2) Participants must withdraw if adverse events (AEs) occur that require their withdrawal from the trial. (3) Participants are unable to complete protocol-specified procedures during the treatment or follow-up phases. (4) Participants can withdraw from the study for any reason. Participants will be asked if they agree to use their data for further analysis and sharing with researchers relevant to this study should they choose to withdraw from the trial.

### 2.5 Interventions

The interventions for both the verum acupuncture group and the sham acupuncture group will be conducted in separate rooms within the outpatient clinic or on the ward. To mitigate treatment bias, two acupuncturists licensed by the Ministry of Health of the People’s Republic of China will administer acupuncture therapy. These practitioners are required to have more than 3 years of acupuncture experience. Prior to the commencement of the trial, the two acupuncturists will undergo professional training, encompassing the study objectives, treatment techniques, and quality assurance measures. Our adherence to the Standard Protocol Items: Recommendations for Interventional Trials (SPIRIT) 2013 statement ensures the incorporation of standardized protocol elements for clinical trials (34).

Participants will undergo acupuncture treatment twice weekly throughout the 8-week trial period, receiving either verum or sham acupuncture. The procedure will utilize sterile, disposable thin filiform needles (25–40 mm long and 0.25 mm in diameter; Hwatuo, Suzhou, China). Both treatment groups will receive 16 sessions over 4 weeks, with two sessions conducted per week. Each session, whether verum or sham acupuncture, will have a duration of approximately 20 min.

#### 2.5.1 Verum acupuncture group

Participants in the verum acupuncture group will receive acupuncture treatment employing the approach of reconciling qi and blood and tonifying heart and mind. This method, derived from literature and expert acupuncture perspectives, involves inserting needles at specific acupuncture points. The acupoints for the verum acupuncture group are as follows:

Treatment will be administered to patients in the supine position at specific acupoints. These include the unilateral points CV17 (Danzhong), CV12 (Zhongwan), and CV6 (Qihai), as well as the bilateral points ST36 (Zusanli) and SP10 (Xuehai). The insertion angle for CV17 (Danzhong) is approximately 10° relative to the skin, with an optimal insertion depth of 12.5 mm. For CV12 (Zhongwan) and CV6 (Qihai), a 0.25 × 40 mm acupuncture needle is inserted to a depth of 25–40 mm. Similarly, ST36 (Zusanli) and SP10 (Xuehai) on both sides are treated with a 0.25 × 40 mm acupuncture needle, inserted to a depth of 20–25 mm.

Patients will receive treatment in prone positions at specific acupoints: GV20 (Baihui), GV16 (Fengfu), BL15 (Xinshu, bilaterally), BL45 (Yixi, bilaterally), HT5 (Tongli, bilaterally), and KI6 (Zhaohai, bilaterally). The insertion angle for GV20 (Baihui) is approximately 10–20° relative to the scalp, with an optimal insertion depth of 5 mm. GV16 (Fengfu) is inserted 12.5–25 mm deep, directed toward the lower jaw, using a 0.25 × 25 mm acupuncture needle. BL15 (Xinshu, bilaterally) and BL45 (Yixi, bilaterally) are inserted at a 10–20° angle relative to the skin, with depths of 7.5–12.5 mm and 12.5–20 mm, respectively, both utilizing 0.25 × 25 mm acupuncture needles. HT5 (Tongli, bilaterally) is inserted 7.5–12.5 mm deep, while KI6 (Zhaohai, bilaterally) is inserted 12.5–20 mm deep, both using 0.25 × 25 mm acupuncture needles. Comprehensive details of the acupoints for the verum acupuncture group are presented in Table 2.

**Table 2:** The details of verum acupoint localization.

Acupoint	Location
CV17 (Danzhong)	In the anterior thoracic region, at the same level as the fourth intercostal space, on the anterior median line.
CV12 (Zhongwan)	On the upper abdomen, 4 B-cun superior to the center of the umbilicus, on the anterior median line.
CV6 (Qihai)	On the lower abdomen, 1.5 B-cun inferior to the center of the umbilicus, on the anterior median line.
SP10 (Xuehai)	On the anteromedial aspect of the thigh, on the bulge of the vastus medialis muscle, 2 B-cun superior to the medial end of the basc of the patella (needled bilaterally)
ST36 (Zusanli)	On the anterior aspect of the leg, on the line connecting ST35 (Dubi) with ST41 (Jiexi), 3 B-cun inferior to ST35 (needled bilaterally)
GV20 (Baihui)	On the head, 5 B-cun superior to the anterior hairline, on the anterior median line.
GV16 (Fengfu)	In the posterior region of the neck, directly inferior to the external occipital protuberance, in the depression between the trapezius muscles.
BL15 (Xinshu)	In the upper back region, at the same level as the inferior border of the spinous process of the fifth thoracic vertebra (T5), 1.5 B-cun lateral to the posterior median line (needled bilaterally)
BL45 (Yixi)	In the upper back region, at the same level as the inferior border of the spinous process of the sixth thoracic vertebra (T6), 3 B-cun lateral to the posterior median line (needled bilaterally)
HT5 (Tongli)	On the anteromedial aspect of the forearm, radial to the flexor carpi ulnaris tendon, 1 B-cun proximal to the palmar wrist crease (needled bilaterally)
KI6 (Zhaohai)	On the medial aspect of the foot, 1 B-cun inferior to the prominence of the medial malleolus, in the depression inferior to the medial malleolus (needled bilaterally)

Prior to needle insertion, the designated areas will be thoroughly disinfected and acupuncture devices will be applied to each location. Subsequently, the acupuncturist will insert a disposable sterile needle into the acupuncture point, adhering to specific position and operation standards. The practitioner will perform appropriate insertion, lifting, and twisting techniques within the patient’s tolerance to elicit the “de qi” sensation, characterized by swelling, soreness, numbness, and heaviness. During the needle retention period, manual manipulation of the needles will occur every 10 min to maintain the “de qi” sensation. Each participant will undergo two 20-min treatment sessions per week, totaling 16 sessions over an 8-week period. Follow-up assessments will be conducted at weeks 8 and 16.

#### 2.5.2 Sham acupuncture group

Patients in the sham acupuncture group will receive treatment at non-meridian and non-acupoint locations without eliciting the sensation of “de qi.” A superficial skin penetration technique will be employed. Several randomized controlled trials of acupuncture have utilized superficial skin penetration at non-acupoints without needle manipulation to avoid inducing the “de qi” sensation as a standard sham acupuncture method (35, 36). The non-meridian and non-acupoint groups will function as controls for the manual puncture and specific acupoint groups. Table 3 provides detailed information on the locations of non-acupoints. The acupuncture procedure and treatment frequency for the sham acupuncture groups will mirror those of the verum acupuncture groups.

**Table 3:** The details of sham acupoint localization (52, 53).

Non-acupoint (needled bilaterally)	Location
Non-acupoint 1	On the anteromedial aspect of the forearm, in the depression ulnar to the flexor carpi ulnaris tendon, the midpoint of the line connecting HT3 (Shaohai) with HT7 (Shaohai).
Non-acupoint 2	On the anteromedial aspect of the forearm, the junction of the upper one fourths and the lower three fourth of the line connecting HT3 (Shaohai) with HT7 (Shaohai).
Non-acupoint 3	On the lateral side of the lower leg, 3 B-cun above the tip of external malleolus, between Stomach Meridian of Foot-Yangming and Gallbladder Meridian of Foot-Shaoyang, at the same level as GB39 (Xuanzhong).
Non-acupoint 4	On the fibular aspect of the leg,3 B-cun inferior to the GB34 (Yanglingquan), On the fibular aspect of the leg,3 B-cun inferior to the GB34, between bladder Meridian of Foot-Taiyang and Gallbladder Meridian of Foot-Shaoyang.
Non-acupoint 5	On the radial side, the midpoint between the medial epicondyle of humerus and the styloid process of the ulna.
Non-acupoint 6	On the thigh,0.8 B-cun medial to the midpoint of the line linking the anterior superior iliac spine and the lateral end of the base of patella (needled bilaterally)
Non-acupoint 7	On the lateral side of the lower leg, 1 cun lateral to ST36 (Zusanli), between the Stomach Meridian of Foot-Yangming and the Gallbladder Meridian of Foot-Shaoyang.
Non-acupoint 8	On the middle of the abdomen, 1 B-cun lateral to ST25 (Tianshu), the midpoint between ST25 and SP15 (Daheng).
Non-acupoint 9	On the middle of the abdomen, 1.2 B-cun lateral to CV12 (Zhongwan).
Non-acupoint 10	On the medial anterior border of the upper arm, the junction of the deltoid and the biceps brachii.
Non-acupoint 11	At the lower border of the medial condyle of the tibia, 1 B-cun anterior and superior to LR7 (Xiguan) of the liver meridian.

### 2.6 Sample size calculation

The sample size calculation was performed using PASS 15.0.5 software (NCSS, LLC). The estimation was based on the change in the 8-week Montreal Cognitive Assessment (MoCA) score post-treatment. Previous studies (17, 18) reported a total effective rate of 13.72% for the 8-week MoCA score in the sham acupuncture group after treatment. We anticipated a total effective rate of 58.33% for the 8-week MoCA score post-treatment in the verum acupuncture group. With a significance level of *α* = 0.05 (two-sided) and a power of 0.9, accounting for a 20% attrition rate, and an equal 1:1 group allocation, a minimum of 25 cases per group were determined necessary. Consequently, this study requires a total of 50 participants.

### 2.7 Outcome measures

#### 2.7.1 Primary indicator

The primary outcome measure is the total effective rate of the 8-week MoCA score. Changes in the MoCA score from baseline to the end of treatment and the follow-up period will assess patients’ cognitive function. The MoCA, a concise cognitive function assessment tool comprising 12 items, was developed by Nasreddine in 2005. It evaluates visuospatial abilities, executive function, short-term memory, language, and orientation to time and place (19). The MoCA test has a maximum score of 30 points, with scores between 21 and 26 indicating mild cognitive impairment. For individuals with 12 or fewer years of education, one point is added to the final score (37). The lower the score, the worse the cognitive function. To evaluate cognitive function, the MoCA score and total improvement rate will be assessed at baseline, at the conclusion of treatment, and during the follow-up period. The score of the MoCA, the absolute changes in scores from baseline to the end of treatment, and the follow-up period will be recorded. The minimum clinically significant difference of MoCA is 1.22 points (38). Improvement rate = [(posttreatment score − pretreatment score)/pretreatment score] × 100%. The curative effect is judged as follows: Significantly effective: Improvement rate≥20%; Effective: 12% ≤ Improvement rate<20%; Invalid: −12% ≤ Improvement rate<12%; Total effective rate according to the formula: (Significantly effective + Effective)/Total cases×100%.

#### 2.7.2 Secondary indicator

The secondary outcomes encompass the Mini-Mental State Examination (MMSE) score, EORTC QLQ-C30 score, and the expression levels of inflammatory cytokines [tumor necrosis factor-*α* (TNF-α), interleukin-2 (IL-2), interleukin-4 (IL-4), interleukin-5 (IL-5), interleukin-6 (IL-6), interleukin-8 (IL-8), interleukin-10 (IL-10), interleukin-17 (IL-17), interleukin-1β (IL-1β), interleukin-12p70 (IL-12p70), interferon-*γ* (IFN-γ), and interferon-α (IFN-α)].

##### 2.7.2.1 MMSE

The MMSE is widely employed as a screening tool to detect mild cognitive impairment through brief assessments (39). This questionnaire encompasses simple tasks across various domains: temporal and spatial orientation, word list repetition, arithmetic operations such as serial subtractions of seven, language comprehension and usage, and fundamental motor skills. A higher score indicates better overall cognitive function, with a score of 27–30 indicating normal function, 21–26 indicating mild impairment, 10–20 indicating moderate impairment, and 0–9 indicating severe impairment. For a respondent with a high-school education and above, a score lower than 24 indicates possible cognitive impairment, and for a respondent with only a primary school education or who is illiterate, the cutoff scores are, respectively, 20 and 17. A lower score indicates a higher degree of cognitive impairment (40). The MMSE will be utilized to evaluate mild cognitive impairment in patients at baseline, upon completion of treatment, and during the follow-up period.

##### 2.7.2.2 European organization for research and treatment of cancer QLQ-C30 (EORTC QLQ-C30)

The EORTC QLQ-C30 is a patient-reported outcome measure comprising 30 items designed to evaluate the health-related quality of life in cancer patients.

The EORTC QLQ-C30 is transformed into 15 scales using a standardized scoring methodology. It encompasses 5 functional scales, 8 symptom scales, an item evaluating financial difficulty, and one global health status scale. The functional scale incorporates physical, role, emotional, cognitive, and social functioning. The symptom scales or individual items comprise fatigue, pain, nausea/vomiting, dyspnea, insomnia, appetite loss, constipation, and diarrhea (41). The summary score is determined using the transformation of all the EORTC QLQ-C30 scale and item scores, however not including global health status and financial impact. For the functional and the global health status, a higher score indicates better health. For the symptom scales, a higher score indicates more symptom burden. The EORTC QLQ-C30 summary score is calculated as the mean of the combined 13 EORTC QLQ-C30 scale and item scores (excluding the global health status and financial impact), with a higher score indicating a better health-related quality of life (42). The EORTC QLQ-C30 score will be evaluated at baseline, upon treatment completion, and during the follow-up period to assess health-related quality of life.

##### 2.7.2.3 Expression of inflammatory cytokines

To investigate the potential mechanisms of acupuncture in patients with triple-negative breast cancer chemotherapy-related cognitive impairment, this study will select 15 patients from each the verum acupuncture group and the sham acupuncture group and collect cytokine data at baseline, at the end of treatment, and during the follow-up period. The equipment utilized for this purpose comprises 12 test kits for cytokines (magnetic particle luminescence). On the day of the cognitive function evaluation, 3 mL blood samples will be drawn from the patients and stored in a serum separation tube. All samples will be centrifuged for 5 min at 1500 RPM. Subsequently, the plasma and serum will be processed, and the supernatants stored at −20°C until analysis. The following blood biomarkers will be measured by experienced technicians blinded to the clinical data: human serum TNF-*α*, IL-2, IL-4, IL-5, IL-6, IL-8, IL-10, IL-17, IL-1β, IL12-P70, IFN-*γ*, and IFN-α. Testing will be conducted in strict accordance with the reagent instructions. Twelve test kits for cytokines (magnetic particle luminescence) will be prepared. The reagent kit will be brought to room temperature prior to the experiment (18–28°C). Standards and quality control solutions will be prepared according to the manufacturer’s instructions. Using immunoassay and multiplex detection techniques to detect cytokine concentrations. Then the samples and standards will be separately pipetted into designated reaction wells. The microplate will then be placed on a magnetic plate and allowed to stand for 1 min. After three repetitions of washing cycles, perform machine testing. All samples will be evaluated in duplicate and averaged. These measures will be assessed at baseline, at the end of treatment, and during the follow-up period. The detailed outcome assessment time points are provided in Table 1.

### 2.8 Safety assessments

The study will meticulously document any adverse events occurring during the treatment period, including their characteristics, timing, severity (mild, moderate, or severe), duration, progression, and outcomes. All adverse events, regardless of relation to the acupuncture cure, are meticulously documented and evaluated by the researchers on the adverse event form at each assessment. The causal relationship between adverse events and acupuncture treatment will be promptly assessed, and the correlation between them will be analyzed (unrelated, possibly related, or definitely related). Appropriate interventions will be implemented immediately to ensure participant safety, and these measures will be recorded. Common adverse events associated with acupuncture include pain, bruising, and localized swelling at the needle insertion sites, which typically resolve spontaneously with rest or compression. Serious cases should to be reported to the Medical Ethics Committee of Beijing Hospital of Traditional Chinese Medicine, Capital Medical University within 24 h and their participation in the trial will be aborted. Upon occurrence of an adverse event, participants will immediately receive appropriate treatment and decide whether to withdraw from the trial at their own discretion. Participants who abort the trial will continue to be investigated and followed up, and the results will be recorded accordingly.

## 3 Data management and quality control

A printed Case Report Form (CRF) will be utilized for clinical data management. The recorded clinical data are considered original and immutable. A designated researcher on the team will maintain custody of the form, with access restricted to authorized researchers. Upon completion of the trial, designated researchers will input the clinical data into a computer system. The sponsor should keep CRF for 5 years after the end of the research. All data will be stored with the principal investigator in a password-protected computer for a period of 5 years. All soft copies of the data will be permanently deleted from the computer once the data storage time is over or at the conclusion of the study. According to the terms of informed consent, unless the subject’s consent is obtained, all the subject’s personal information is confidential and will not be disclosed to the public. The results of this study will be published in peer-reviewed journals and posters or oral presentations at relevant conferences. All data will be available beginning 3 months and ending 3 years after publication of the results. The trial data will be available from the corresponding author upon reasonable request.

## 4 Statistical analysis

The statistical analysis will be conducted using Microsoft Excel 2016 and SPSS 25.0 statistical software (IBM Corporation, Armonk, NY, United States). An independent statistician, blinded to group assignment, will perform the statistical analysis. All data will be analyzed according to the intention-to-treat (ITT) protocol, regardless of adherence, provided participants receive at least one acupuncture treatment session and complete a baseline assessment of the primary outcome. Missing values will be imputed using the last observation carried forward method. The per-protocol (PP) population, comprising all randomized patients who completed more than 80% of the treatment procedure, will also be analyzed. All statistical tests will be two-sided, with a *p* value of <0.05 considered statistically significant. Categorical variables will be reported as medians (interquartile ranges), while continuous variables with normal distributions will be presented as means ± standard deviations. The chi-square test or Fisher’s exact test will be employed to compare dichotomous data from baseline demographic and clinical information. Measurement data include various scale scores and indicators of inflammatory cytokine expression levels. For normally distributed variables, an independent samples t-test will be used for between-group comparisons, and a paired samples t-test for within-group comparisons before and after therapy. Comparisons at multiple time points will be made using repeated-measures analysis of variance. The Mann–Whitney U test will be utilized for non-normally distributed variables in intergroup comparisons to analyze changes in efficacy outcomes. The comparison of multiple time points will be based on the generalized estimating equation (GEE). Multiple linear regression analysis will be employed to examine correlations between clinical efficacy and inflammatory cytokines.

## 5 Trial status

The Medical Ethics Committee of Beijing Hospital of Traditional Chinese Medicine, Capital Medical University, China, granted approval for this study on 9 January 2024. Currently, the trial is in the recruitment phase, which is scheduled to continue until May 2026. As of now, 20 patients have been enrolled, with the initial participant joining on 19 February 2024.

## 6 Discussion

CRCI represents a significant and prevalent issue for breast cancer survivors, adversely affecting their quality of life (32). While CRCI in breast cancer patients undoubtedly warrants increased attention, there is currently no established principle or guideline for its management. This single-blind, randomized, controlled trial aims to evaluate the efficacy and safety of acupuncture in treating CRCI in TNBC patients, while also exploring the potential mechanisms underlying this treatment approach. Acupuncture, a traditional Chinese medical therapy, has emerged as a promising intervention for mild cognitive impairment. Multiple studies have demonstrated acupuncture’s efficacy in enhancing cognitive function across various pathological conditions (43–45). Our previous research, employing the principle of “reconciling qi and blood, tonifying heart and mind,” has substantiated the effectiveness and safety of acupuncture for breast cancer patients experiencing CRCI (17, 46). This study specifically focuses on TNBC patients due to their higher incidence of CRCI and its consequential impact on quality of life. To the best of our knowledge, no studies have yet investigated the efficacy of acupuncture in managing CRCI among TNBC patients.

Chemotherapy has been observed to elevate the levels of inflammatory mediators such as IL-1β, TNF-*α*, and COX-2, while simultaneously reducing the levels of the anti-inflammatory cytokine IL-10 (47). These inflammatory factors can induce neuroinflammation and contribute to cognitive impairment. Given the crucial role of inflammatory factors in the development of CRCI, this study also measured serum inflammatory factor levels. This approach allows for an exploration of the therapeutic mechanism of acupuncture in treating CRCI.

This randomized, sham-acupuncture-controlled study was primarily designed to investigate the efficacy and safety of acupuncture in managing CRCI among TNBC patients and provide physicians with evidence-based guidelines for treating CRCI. Furthermore, this trial aims to explore the mechanisms through which acupuncture may exert its therapeutic effects in breast cancer patients with CRCI, potentially offering new insights into its therapeutic efficacy.

Nevertheless, the current study design has certain limitations. In this trial, the sham acupuncture group uses a non-meridian and non-acupoint needling approach where shallow needling is performed on superficial skin to minimize stimulation and avoid inducing the arrival of qi sensation. However, it cannot be guaranteed that sham acupuncture will have no therapeutic effect on the disease. Recent studies on the non-penetration of the sham needles have yielded inconsistent results. Some researchers contend that superficial needling can produce specific acupuncture effects, resulting in false negatives (48). Studies have shown that the responses to tactile stimulation such as skin compression or prickling sensations may elicit certain physiological responses in the body and activate the somatosensory system and relevant brain regions and thereby produce specific effects (49, 50). However, recent research suggests that skin-penetrating sham needles can be used as inert placebo controls in randomized controlled trials (51). In conclusion, the potential for physiological effects arising from the control group’s design cannot be disregarded. Previous randomized controlled trials have demonstrated that superficial insertion may elicit physiological responses that alleviate symptoms. Although this study will select sham points to avoid meridians and acupoints, the possibility of distinct physiological effects in the control group cannot be entirely eliminated. Another primary limitation is the inability to blind the treating therapists. Achieving true double-blind conditions in acupuncture trials is practically unfeasible, as acupuncturists manage participants’ perceptions of needling. Consequently, differentiating between the actual impact of acupuncture and placebo or physiological effects remains challenging. The potential for bias due to the lack of blinding cannot be excluded. Additionally, in the context of a chronic disease such as CRCI, a 6-week assessment period may be inadequate for gaining a thorough understanding. To draw definitive conclusions regarding the long-term impact of acupuncture interventions, additional real-world studies involving extended follow-up periods are essential.
